# MicroRNA-3935 promotes human trophoblast cell epithelial-mesenchymal transition through tumor necrosis factor receptor-associated factor 6/regulator of G protein signaling 2 axis

**DOI:** 10.1186/s12958-021-00817-x

**Published:** 2021-09-07

**Authors:** Meiyuan Jin, Shouying Xu, Jiayong Li, Yingyu Yao, Chao Tang

**Affiliations:** 1 National Clinical Research Center for Child Health of the Children’s Hospital, Zhejiang University School of Medicine, No. 3333, Binsheng Rd, Hangzhou, 310052 China; 2 Department of Obstetrics, Tongde Hospital of Zhejiang Province, Hangzhou, 310012 China; 3 Department of Ophthalmology, Hangzhou Traditional Chinese Medicine Hospital Affiliated to Zhejiang Chinese Medical University, Hangzhou, 310007 China

**Keywords:** miR-3935, TRAF6, RGS2, Trophoblast, EMT

## Abstract

**Background:**

Insufficient migration and invasion during trophoblast epithelial-mesenchymal transition (EMT) results in the occurrence and development of preeclampsia (PE), and our previous study has screened 52 miRNAs, whose expression levels are altered in the placental samples from PE patients, compared with the normal group. Among those, miR-3935 is one of the miRNAs being most significantly down-regulated, indicating its involvement in PE. However, the exact effect and molecular mechanisms remain unknown.

**Methods:**

In the present study, we investigate the roles and underlying mechanisms of miR-3935 in trophoblast EMT by use of the human extra-villous trophoblast cell line HTR-8/SVneo as well as human placental tissues and maternal blood samples obtained from 15 women with normal pregnancies and 15 women with PE. Experimental methods include transfection, quantitative reverse transcription-PCR (qRT-PCR), western blot, immunofluorescence staining, dual-luciferase assays, in vitro invasion and migration assays, RNA-Seq analysis, bisulfite sequencing and immunohistochemistry staining.

**Results:**

MiR-3935 expression is significantly decreased in both placentas and peripheral blood specimens of PE, and functionally, miR-3935 promotes EMT of trophoblast cells. Mechanistically, TRAF6 is identified to be a direct target of miR-3935 and TRAF6 exerts its negative effect on EMT of trophoblast cells by inhibition of RGS2, which down-regulates the methylation status of promoter of *CDH1* gene that encodes E-Cadherin protein through induction of ALKBH1, resulting in increase of E-Cadherin and subsequently insufficient trophoblast EMT.

**Conclusions:**

Together these results uncover a hitherto uncharacterized role of miR-3935/TRAF6/RGS2 axis in the function of human trophoblasts, which may pinpoint the molecular pathogenesis of PE and may be a prognostic biomarker and therapeutic target for such obstetrical diseases as PE.

## Introduction

Preeclampsia (PE), a relatively common pregnancy disorder, is still one of the leading causes of maternal and neonatal morbidity and mortality worldwide, and in the worst cases, may threaten the survival of both mother and baby [1–3]. However, its etiology, pathogenesis and treatment remain unclear, and the changes in patient conditions appear to be different and complex and thus difficult to predict. As a result, there is a particularly urgent need to clarify the pathogenesis of PE and to discover prognosis-related indicators. Termination of pregnancy to deliver the placenta appears to be the most effective method to control PE, demonstrating that PE is a placenta-borne disease [4, 5]. At present, increasing evidence has been found in support of the theory of trophoblast superficial implantation, whereby abnormal migration and invasion during trophoblastic epithelial mesenchymal transition (EMT) brings about the occurrence and development of PE [5–7].

EMT is a biologic process in which the polarized epithelial cells that normally interacts with basement membrane via its basal surface undergo multiple biochemical changes, including loss of their cell polarity, tight cell–cell junctions, and adhesion characteristics, and acquirement of migratory and invasive potential, to become cells with mesenchymal cell morphology and properties [8, 9]. Previous studies reported that following the earliest stages of embryogenesis, the implantation of the embryo and the initiation of placenta formation are both associated with an EMT that involves the parietal endoderm [8, 10, 11]. In particular, the invasion of the endometrium on maternal uterine wall and the subsequent proper anchoring of the placenta by trophoblast cells involve a series of changes in trophoblast cell morphology that are completed by placental trophoblast cell EMT, whereas shallow placental implantation and defective spiral artery conversion due to impaired invasion are implicated in the etiology of major placental pathologies, such as PE [6, 7]. Therefore, we speculate that PE may be associated to a certain extent with dysfunctions in the EMT of placental trophoblast cells.

MicroRNAs (miRNAs), which comprise a large family of small single-strand non-coding endogenous RNAs, have emerged as key posttranscriptional regulators of gene expression [12, 13]. In mammals, miRNAs are predicted to control the activity of more than 60% of all protein-encoding genes and regulate protein synthesis by base-pairing to 3’-UTR of target mRNAs. Recent evidence suggests that miRNAs are involved in almost every cellular process, including EMT [14, 15].

Our previous study has revealed that 22 miRNAs are up-regulated and 30 miRNAs are down-regulated in the placental samples from PE patients, and among the aberrantly expressed miRNAs, miR-20b is one of the miRNAs being most significantly up-regulated, which inhibits trophoblast cell migration and invasion by targeting MMP-2 [16]. However, the effect of the significantly down-regulated miRNAs on PE and their mechanisms are yet to emerge. In the present study, we investigate the potential role of miR-3935, whose expression is markedly inhibited in PE group, in trophoblastic EMT. We find that miR-3935 expression is decreased in placentas and peripheral blood specimens of PE. Functionally, we demonstrate that miR-3935 can promote trophoblast cells EMT. Mechanistically, TRAF6 is identified to be a direct target of miR-3935 and TRAF6 exerts its negative effect on EMT of trophoblast cells by suppression of RGS2, which promotes the methylation status of promoter of *CDH1* gene that encodes E-Cadherin protein through down-regulation of the expression of the DNA demethylase ALKBH1, resulting in inhibition of E-Cadherin and subsequently initiation of EMT progression.

## Materials and methods

### Cell culture

The extra-villous trophoblast cell line HTR-8/SVneo was purchased from the American Type Culture Collection (ATCC, Manassas, USA) and maintained in RPMI 1640 medium (Life Technologies) supplemented with 10% fetal bovine serum (FBS, Life Technologies). Human embryonic kidney 293 T cells (HEK293T) were obtained from ATCC and maintained in Dulbecco’s modified Eagle’s medium (DMEM, Invitrogen) containing 10% FBS. The cells were incubated at 37 °C with 5% CO_2_.

### Transfection

The miR-3935 mimics (5’-UGUAGAUACGAGCACCAGCCAC-3’), miR-3935 inhibitor (5’-GUGGCUGGUGCUCGUAUCUACA-3’) and their corresponding negative control (NC, 5’-UUUGUACUACACAAAAGUACUG-3’) were synthesized by RiboBio (Guangzhou, China). Human RGS2 overexpression plasmid (pcDNA3-Flag-hRGS2) was constructed by Dr. Shouying Xu, and *RGS2* siRNA for silencing human RGS2 were purchased from RiboBio (Guangzhou, China). Cell transfection was performed using Lipofectamine 2000 (Invitrogen, Carlsbad, CA, USA) according to the manufacturer’s instructions. The cells were treated for further experiments 48 h (for plasmid) or 72 h (for siRNA) after transfection.

### RNA extraction and quantitative reverse transcription-PCR (qRT-PCR)

Total RNA was isolated from placentas, peripheral blood specimens and HTR-8/SVneo cells using the TRIzol reagents (TAKARA, Dalian, China) following the manufacturer’s instructions. For the detection of miR-3935, qRT-PCR assays were performed using the TaqMan miRNA Assay (Life Technologies) following the manufacturer’s instructions. For detection the mRNA levels of *RGS2*, *E-Cadherin*, *TRAF6* and *CTR9*, 1 μg of total RNA was reverse transcribed using Script™ cDNA Synthesis Kit (Bio-Rad Laboratories), then performed on an Applied Biosystems 7900HT cycler using SuperReal PreMix Plus (Tiangen, China). U6 functioned as the normalization control in the expression analysis of miR-3935 and *GAPDH* was used as the normalization control in the expression analysis of *RGS2*, *E-Cadherin*, *TRAF6* and *CTR9*, respectively. The relative expression of RNAs was calculated using the 2^−ΔΔCt^ method. Each reaction was conducted in triplicate.

### Western blot

Western blots were performed using standard protocols. Cell lysates were prepared using RIPA buffer. Protein samples were subjected to SDS–polyacrylamide gel electrophoresis (SDS-PAGE) and transferred onto a PVDF membrane (Millipore). The membrane was blocked with 5% non-fat milk. The membrane was then incubated with anti-E-Cadherin antibody (1:1000, bs-10009R, Bioss, Beijing, China), anti-Vimentin antibody (1:1000, bs-0756R, Bioss, Beijing, China), anti-TRAF6 antibody (1:1000, bs-1184R, Bioss, Beijing, China), anti-N-Cadherin antibody (1:1000, AF5237, Beyotime, Shanghai, China), anti-RGS2 antibody (1:1000, sc-100761, Santa Cruz), anti-Flag antibody (1:1000, M185-3, MBL, Beijing, China) or anti-αTubulin antibody (1:5000, AF5012, Beyotime, Shanghai, China) at 4 °C overnight, followed by incubation with HRP-anti-rabbit or HRP-anti-mouse secondary antibody at room temperature for 1 h. Signals were detected using an ECL Kit (P0018AS, Beyotime, Shanghai, China) according to the manufacturer’s protocol.

### Immunofluorescence staining

Immunofluorescence staining was performed on chamber slides (Nalge Nunc International, Naperville, IL). miRNA-3935 mimics or negative control (NC)-transfected HTR-8/SVneo cells were fixed in ice-cold 4% paraformaldehyde (PFA) and permeabilized with 0.1% TritonX-100 in PBS (PBST). After incubation with blocking buffer (1% bovine serum albumin, BSA), cells were incubated with primary antibody against E-Cadherin and were subsequently incubated with Alexa 488-conjugated secondary antibody (Life Technologies). Nuclei were counterstained with 4',6-diamidino-2-phenylindole (DAPI). Slides were analyzed by a laser scanning microscope (Zeiss).

### Dual-luciferase assays

A cDNA fragment of the *TRAF6* 3’-UTR mRNA containing the seed sequence of the miR-3935-binding site or a mutated binding site was cloned into the pmirGLO dual-luciferase vector (Promega, Madison, WI, USA). The constructed dual-luciferase vector was co-transfected with 10 pmol of miR-3935 mimics, miR-3935 inhibitor or corresponding negative control (NC) into HEK293T cells or HTR-8/SVneo cells. The cells were harvested and lysed 24 h later, and the luciferase activity was measured by the Dual-Luciferase Assay System (Promega) as per the manufacturer’s instructions.

### In vitro* invasion and migration assays*

For wound healing assay, HTR-8/SVneo cells were transfected with miR-3935 mimics, miR-3935 inhibitor or corresponding negative control (NC) and RGS2, control vector, siRGS2 or control siRNA (siCon), and seeded in 6-well plates. After 48 h, when the cells reached 80% confluence, scratch wounds were wounded with a 10 μl pipette tip, then cells were washed three times with PBS, and wound gaps were subsequently imaged and calculated by ImageJ software (NIH, Bethesda, MD, USA) at 48 h.

For invasion assay, the invasive ability of HTR-8/SVneo cells was evaluated using the Transwell Matrigel invasion assay, as previously described [17]. The number of invaded cells was calculated by counting five random views under the microscope at 48 h.

### RNA-Seq analysis

A minimum of 3 μg of total RNA was oligo (dT) selected using the Dynabeads mRNA purification kit (Invitrogen). The mRNA isolated from total RNA was fragmented into short fragments with a fragmentation buffer (Ambion). Double-stranded cDNA was synthesized with these short fragments as templates. The cDNA was end-repaired, ligated to Illumina adapters, size selected on agarose gel (approximately 250 bp) and PCR amplified. The cDNA library was sequenced on an Illumina HiSeq 2000 sequencing platform (Berry Genomics). The gene expression levels for each transcript were estimated as the number of reads per kilo-base of exon model per million mapped reads (RPKM) using only uniquely mapped reads in exonic regions. A gene is considered significantly differentially expressed if its expression differs between samples from the two groups, NC and miR-3935 mimics, with the log Fold Change > 1 or < -1 and the *p* value < 0.05 as calculated by Cufflinks.

### Bisulfite sequencing

Bisulfite sequencing was performed to determine the methylation pattern of the *CDH1* gene promoter. Briefly, genomic DNA from human placental tissues was extracted using the DNeasy Blood and Tissue kit (QIAGEN). Sodium bisulfite treatment of genomic DNA was performed using an EpiTect Bisulfite kit (QIAGEN), according to the manufacturer's protocol. Following bisulfite treatment, the promoter region of the *CDH1* gene was amplified by PCR using the following primers: 5’-TGGCTCATGCCTGTAATCCCAG-3’ (forward) and 5’-CCTCGCAAGTCAGGGGATCCG-3’ (reverse). The PCR products were cloned into the pT7 blue vector (Novagen), and 10 randomly selected clones were sequenced using the T7 and M13 universal primers.

### Tissue samples

Placental tissues (1 cm*1 cm*1 cm) and maternal blood samples (5 ml) were obtained from 15 women with normal pregnancies [age (years): 28.6 ± 2.6, height (cm): 161.3 ± 3.3, weight (kg): 76.1 ± 3.1, gestational age at delivery (weeks): 39.1 ± 0.8] and 15 women with PE [age (years): 30.7 ± 3.0, height (cm): 162.1 ± 5.0, weight (kg): 75.7 ± 2.8, gestational age at delivery (weeks): 38.7 ± 1.3] at Department of Obstetrics, Tongde Hospital of Zhejiang Province. The samples were obtained between 29 and 40 weeks of gestation from Jan to Dec of 2020. Patients with PE were defined as having systolic and diastolic blood pressure > 140 and 90 mmHg, respectively, measured at least 6 h apart plus proteinuria ≥ 300 mg/24 h or > 1 + by dipstick test. Clinical states of known pathogenesis which could possibly interfere with values of studied parameters were excluded. Exclusion criteria were also uterine contractions and premature rupture of amniotic membranes. To get similar clinical condition with PE, control group with similar gestational age were collected. All experimental protocols were approved by the Ethics Committee of Tongde Hospital of Zhejiang Province and all studies were performed in accordance with the ethical guidelines of the Ethics Committee of the Tongde Hospital of Zhejiang Province. Informed consent was obtained from all patients.

### Immunohistochemistry staining

Immunohistochemistry staining was performed using the Histostain-Plus Kit (Kangwei Reagents, Beijing, China) as per the manufacturer’s instructions. Briefly, paraffin-embedded placental tissue Sects. (4 μm) were deparaffinized and rehydrated in xylene and a graded series of ethanol. After antigen retrieval, tissue sections were then incubated with 3% H_2_O_2_ in methanol to quench endogenous peroxidase followed by sequential incubation including with normal goat serum for 30 min, with control mouse IgG (sc-2025, Santa Cruz, CA, USA) and primary antibody against RGS2 at 4 °C overnight, and with HRP-labeled secondary antibody (Life Technologies) for 30 min. The diaminobenzidine (DAB) solution was used for development of color, and the sections were counterstained with hematoxylin. RGS2 expression was scored based on the proportion of cells showing RGS2 immunostaining across 3 non-adjacent fields in each sample using the following criteria: 0 (no cell positive); 1 (< 50% cells weakly positive); 2 (< 50% cells intensely staining); 3 (≥ 50% cells weakly positive); 4 (≥ 50% cells intensely staining).

### Statistical analysis

Statistical analysis was performed using GraphPad Prism 5.0 (GraphPad Software, Inc., San Diego, CA, USA). Differences were analyzed with the Student’s t-test between two groups or with one-way ANOVA among multiple groups. Correlation analyses were analyzed with a Pearson analysis. Statistical significance was assessed at *p* < 0.01.

## Results

### MiR-3935 is down-regulated in preeclamptic placenta

**Fig. 1:**
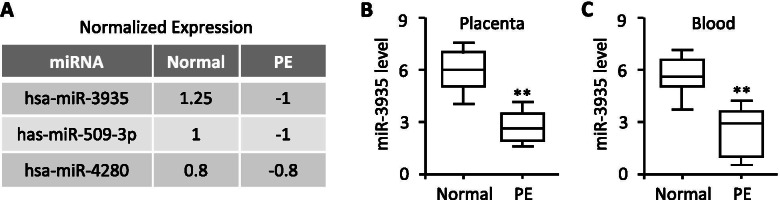
miR-3935 is down-regulated in preeclamptic placentas as and peripheral blood specimens. **A** A table showing the three candidate miRNAs whose expression levels were significantly down-regulated in placentas samples from three patients with PE, compared with those of three women with normal pregnancies. **B** qRT-PCR analysis for miR-3935 levels in preeclamptic placentas (*n* = 10) and normal pregnancies (*n* = 10). **C** qRT-PCR analysis for miR-3935 levels in peripheral blood specimens from patients (n = 10) with PE compared with that from women (*n* = 10) with normal pregnancies. **p < 0.01 versus normal group (Normal)

To determine the potential involvement of miRNAs in the pathogenesis of preeclampsia (PE), we previously performed a miRNA microarray on placental samples from three PE patients and three women with normal pregnancies [16]. Our data revealed that compared with the normal group, 30 miRNAs were down-regulated in the PE group [16], such as miR-3935, miR-509-3p and miR-4280, among which miR-3935 was one of the miRNAs being most significantly down-regulated (Fig. 1A). Given that miR-3935 is involved in male diseases such as prostate cancer [18] while its effect on such female reproductive diseases as PE remains unknown, we thereby chose miR-3935 for further research.

The down-regulated expression trend of miR-3935 obtained from the miRNA microarray assay was further validated by quantitative real-time RT-PCR (qRT-PCR) in placentas and peripheral blood specimens. Results revealed that miR-3935 level was significantly lower in placentas from patients with PE than those with normal pregnancies (Fig. 1B). Similarly, miR-3935 level was also obviously down-regulated in peripheral blood specimens from patients with PE compared with those with normal pregnancies (Fig. 1C). Thus, these results provided sufficient evidence that miR-3935 was prominently down-regulated in PE, suggesting that miR-3935 is involved in PE.

## Transcriptome analysis of miR-3935-regulated genes

**Fig. 2:**
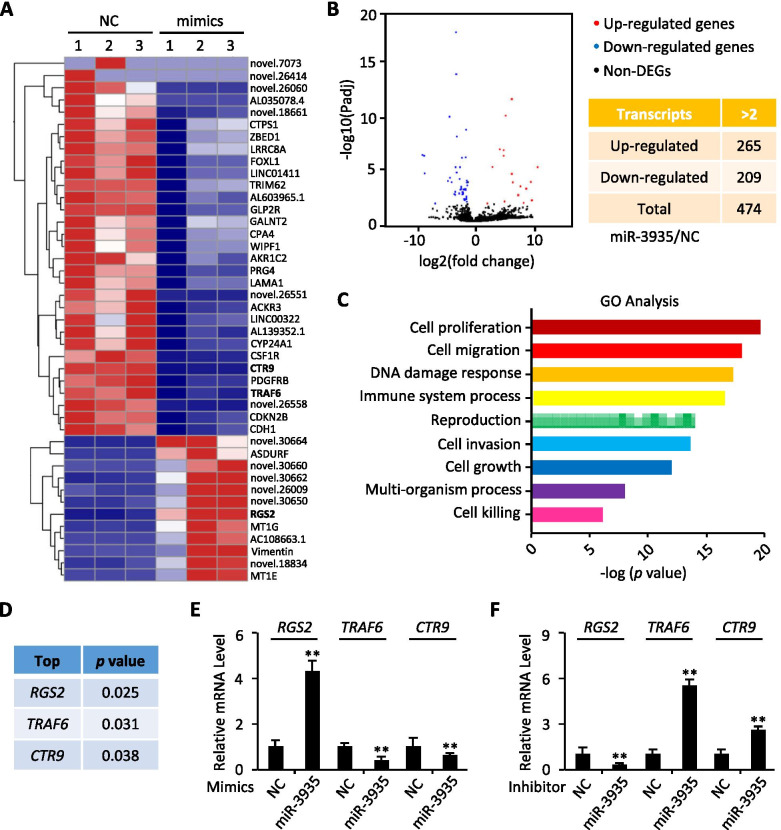
Transcriptome analysis of miR-3935-regulated genes. **A** A heat-map of normalized expression levels of the altered genes measured by RNA-seq comparing negative miRNA control-transfected (NC) and miR-3935 mimics-transfected HTR-8/SVneo cells. Blue indicates low expression levels; red indicates high expression levels. **B** A volcano plot showing the altered genes in the presence of miR-3935 mimics. The number of genes is summarized in the table. **C** GO and pathway analysis of miR-3935-regulated genes measured by RNA-seq comparing negative miRNA control-transfected and miR-3935 mimics-transfected HTR-8/SVneo cells. **D** A table showing the three representative candidate genes, whose expression levels were significantly altered by miR-3935 mimics. **E** qRT-PCR analysis for *RGS2*, *TRAF6* and *CTR9* levels in miR-3935 mimics-transfected or negative miRNA control-transfected (NC) HTR-8/SVneo cells. **F** qRT-PCR analysis for *RGS2*, *TRAF6* and *CTR9* levels in miR-3935 inhibitors-transfected or negative miRNA control-transfected (NC) HTR-8/SVneo cells. **p < 0.01 versus mimics negative control (NC) or inhibitor negative control (NC); data represent the mean ± SD of three independent experiments

To gain insights into the molecular mechanisms underlying the potential role of miR-3935 in PE, RNA-seq was performed in HTR-8/SVneo cells, a first-trimester human extravillous trophoblasts (EVTs)-derived cell line [19], transfected with miR-3935 mimics or negative control (NC). A total of 474 transcripts (log Fold Change > 1 or < -1) were significantly changed upon exposure to miR-3935 mimics, among which 265 were up-regulated and 209 were down-regulated (Fig. 2A,B), respectively, indicating that miR-3935 profoundly altered the transcriptome. Gene ontology (GO) and pathway analysis suggested that gain-of-function of miR-3935 affected many important processes in cell biology, including cell migration and cell invasion, the cell biological behavior altered during EMT progression (Fig. 2C). Among the down-regulated genes, two top candidate target genes with the largest fold-changes were TNF receptor associated factor 6 (*TRAF6*) and CTR9, and based on the lowest *p* value of all the up-regulated genes, *RGS2*, known as a multifunctional regulator of G-protein signaling [20], ranked No.1 (Fig. 2A,D). In addition, some EMT-associated genes [9, 21], such as *CDH1* (*E-Cadherin*) and *Vimentin* were also identified (Fig. 2A). Since RGS2 was identified to be involved in EMT during prostate cancer progression [22], we thereby speculated that miR-3935 might participate in trophoblast cells EMT through up-regulation of *RGS2* transcription. qRT-PCR results confirmed that *RGS2* mRNA expression was elevated in miR-3935 mimics-expressing cells but was decreased in miR-3935 inhibitor-expressing cells, compared with NC, respectively (Fig. 2E,F). Conversely, the other two top-ranked candidate targets, *TRAF6* and *CTR9*, were inhibited or significantly induced by miR-3935 mimics or miR-3935 inhibitor, respectively, which was consistent with the RNA-seq data (Fig. 2A,E,F).

### MiR-3935 regulates RGS2 expression by targeting TRAF6

**Fig. 3:**
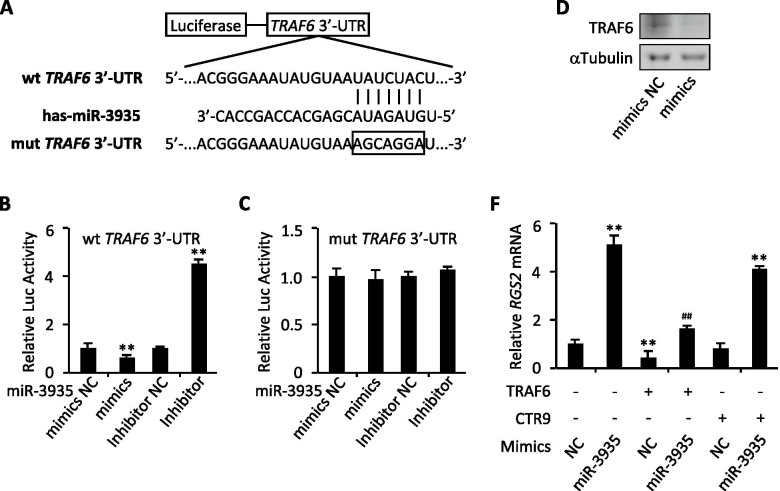
TRAF6 is a direct target of miR-3935. **A** The putative binding site of miR-3935 and RGS2 is shown. **B** Luciferase assay of HEK293T cells co-transfected with firefly luciferase constructs containing the *TRAF6* wild-type 3’-UTRs and miR-3935 mimics, mimics NC, miR-3935 inhibitor or inhibitor NC. **C** Luciferase assay of HEK293T cells co-transfected with firefly luciferase constructs containing the *TRAF6* mutated 3’-UTRs (mut) and miR-3935 mimics, mimics NC, miR-3935 inhibitor or inhibitor NC. **D** Western blot analysis for TRAF6 in miR-3935 mimics or mimics NC-transfected HTR-8/SVneo cells. **E** qRT-PCR analysis for *RGS2* in HTR-8/SVneo cells co-transfected with TRAF6, CTR9 and miR-3935 mimics or mimics NC. **p < 0.01 versus mimics control (NC), inhibitor control (NC), or mimics control without TRAF6 or CTR9 (NC, TRAF6 -, CTR9 -); ##*p* < 0.01 versus mimics control with TRAF6 and without CTR9 (NC, TRAF6 + , CTR9 -); data represent the mean ± SD of three independent experiments

Given the above results obtained, bio-informatic tools were further used to predict the possibility that the three top candidates were targets of miR-3935. Interestingly, only *TRAF6* rather than *RGS2* or *CTR9* could bind to miR-3935, suggesting a potential direct interaction between miR-3935 and *TRAF6* (Fig. 3A). Thus, *TRAF6* was selected for further investigation. To experimentally validate whether *TRAF6* was a direct target of miR-3935, we performed a dual-luciferase reporter assay with the luciferase vectors containing the wild-type *TRAF6* 3’-UTR mRNA fragment that was predicted to bind with miR-3935 or the *TRAF6* 3’-UTR mutant mRNA fragment that was presumed to prevent miR-3935 binding, respectively. Our results revealed that overexpression of miR-3935 mimics decreased the luciferase activity of wild-type *TRAF6* 3’-UTR (wt *TRAF6* 3’-UTR), whereas knockdown of miR-3935 using miR-3935 inhibitor markedly induced luciferase activity (Fig. 3B). However, co-transfection of miR-3935 mimics or miR-3935 inhibitor with *TRAF6* 3’-UTR mutant (mut *TRAF6* 3’-UTR), exhibited no significant change in their luciferase activities, compared with their NC group, respectively (Fig. 3C). Moreover, obviously down-regulated TRAF6 protein expression was observed in the presence of miR-3935 mimics (Fig. 3D). Though RGS2 was not a direct target of miR-3935, its mRNA expression could be altered by overexpression of miR-3935 mimics or inhibition of miR-3935 (Fig. 2E,F). Intriguingly, overexpression of *TRAF6* but not *CTR9* restored RGS2 expression induced by miR-3935 mimics (Fig. 3F), indicating miR-3935 regulates RGS2 expression through silencing of *TRAF6*. Taken together, *TRAF6* is a direct target of miR-3935, and miR-3935 negatively regulates *TRAF6* expression, resulting in up-regulation of RGS2 expression.

### MiR-3935 mediates trophoblast cells EMT through RGS2

**Fig. 4:**
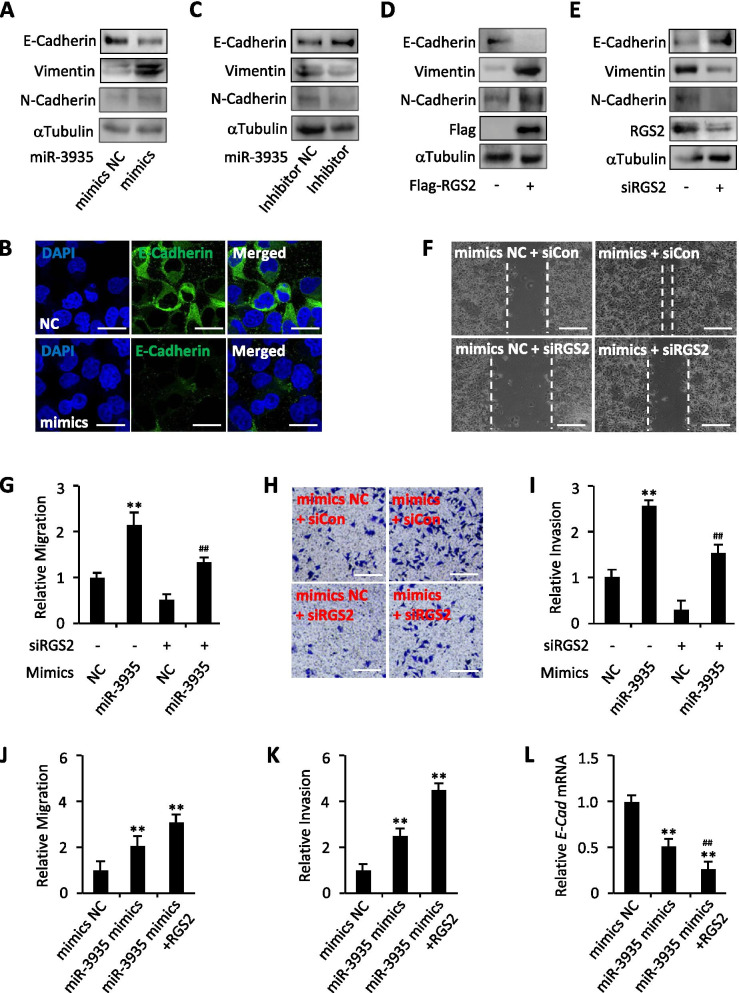
RGS2 exerts the effect of miR-3935 on trophoblast cells EMT. **A** Western blot analysis for E-Cadherin, Vimentin and N-Cadherin expression levels in miR-3935 mimics-transfected or negative control-transfected (NC) HTR-8/SVneo cells. **B** Immunofluorescent staining of E-Cadherin-derived signal in HTR-8/SVneo cells transfected with miR-3935 mimics or negative control (NC). Nuclei were stained with DAPI. **C** Western blot analysis for E-Cadherin, Vimentin and N-Cadherin expression levels in miR-3935 inhibitor-transfected or negative control-transfected (NC) HTR-8/SVneo cells. **D** Western blot analysis for E-Cadherin, Vimentin and N-Cadherin expression levels in Flag-RGS2-transfected or Flag-tagged control vector-transfected (Flag-RGS2, -) HTR-8/SVneo cells. **E** Western blot analysis for E-Cadherin, Vimentin and N-Cadherin expression levels in RGS2 siRNA-transfected (siRGS2) or control siRNA-transfected (siRGS2, -) HTR-8/SVneo cells. **F** Wound healing assay in HTR-8/SVneo cells transfected with miR-3935 mimics or mimics negative control (mimics NC) and RGS2 siRNA (siRGS2) or control siRNA (siCon). **G** Statistical analysis for (**F**). **H** Transwell assay was performed to monitor cell invasion in HTR-8/SVneo cells transfected with miR-3935 mimics or mimics NC and siRGS2 or siCon. **I** Statistical analysis for (**H**). **J** Statistical analysis for migration of HTR-8/SVneo cells co-transfected with miR-3935 mimics or mimics NC and RGS2. **K** Statistical analysis for invasion of HTR-8/SVneo cells co-transfected with miR-3935 mimics or mimics NC and RGS2. **L** qRT-PCR analysis for *E-Cadherin* in HTR-8/SVneo cells co-transfected with miR-3935 mimics or mimics NC and RGS2. **p < 0.01 versus mimics negative control (NC) with siCon or mimics negative control (NC) without RGS2; ##*p* < 0.01 versus miR-3935 mimics with siCon; data represent the mean ± SD of three independent experiments; bar, 10 μm in (**B**), 80 μm in (**F**), 100 μm in (**H**)

Next, we wanted to determine whether miR-3935 affects trophoblast cells EMT. Overexpression of miRNA-3935 decreased E-Cadherin protein expression while increased Vimentin and N-Cadherin protein expression (Fig. 4A), which is consistent with the RNA-Seq data (Fig. 2A), and the absence of E-Cadherin upon exposure to miRNA-3935 mimics was further determined by immunofluorescent staining (Fig. 4B), suggesting miRNA-3935 promotes trophoblast cells EMT. On the other hand, inhibition of miRNA-3935 by inhibitor up-regulated E-Cadherin protein expression but down-regulated that of Vimentin and N-Cadherin (Fig. 4C). Likewise, overexpression of Flag-RGS2 obviously decreased E-Cadherin protein levels and increased protein levels of Vimentin and N-Cadherin (Fig. 4D), whereas knockdown of RGS2 using RGS2 siRNA (siRGS2) resulted in up-regulation of E-Cadherin protein levels as well as down-regulation of protein levels of Vimentin and N-Cadherin (Fig. 4E), providing with the evidence that RGS2 also directs trophoblast cells EMT. Since RGS2 correlated with miR-3935 expression (Fig. 2E,F), we next sought to determine whether RGS2 participated in miR-3935 mediated trophoblast cells EMT. To this end, we silenced RGS2 in the presence of miR-3935 mimics in HTR-8/SVneo cells and explored the alterations of migration and invasion. Wound healing assays and transwell assays exhibited that inhibition of RGS2 by RGS2 siRNA not only reduced HTR-8/SVneo cells migratory and invasive capacities but also significantly reversed the cell migration and invasion that were enhanced by miR-3935 mimics (Fig. 4F-I). Conversely, overexpression of RGS2 further promoted trophoblast cells migration and invasion induced by miR-3935 mimics (Fig. 4J,K) and significantly declined the mRNA expression of *E-Cadherin*, compared with that by miR-3935 mimics alone (Fig. 4L). Thus, miR-3935 enhances trophoblast cells EMT through RGS2.

### RGS2 promotes trophoblast cells EMT by regulation of CDH1 promoter methylation

**Fig. 5:**
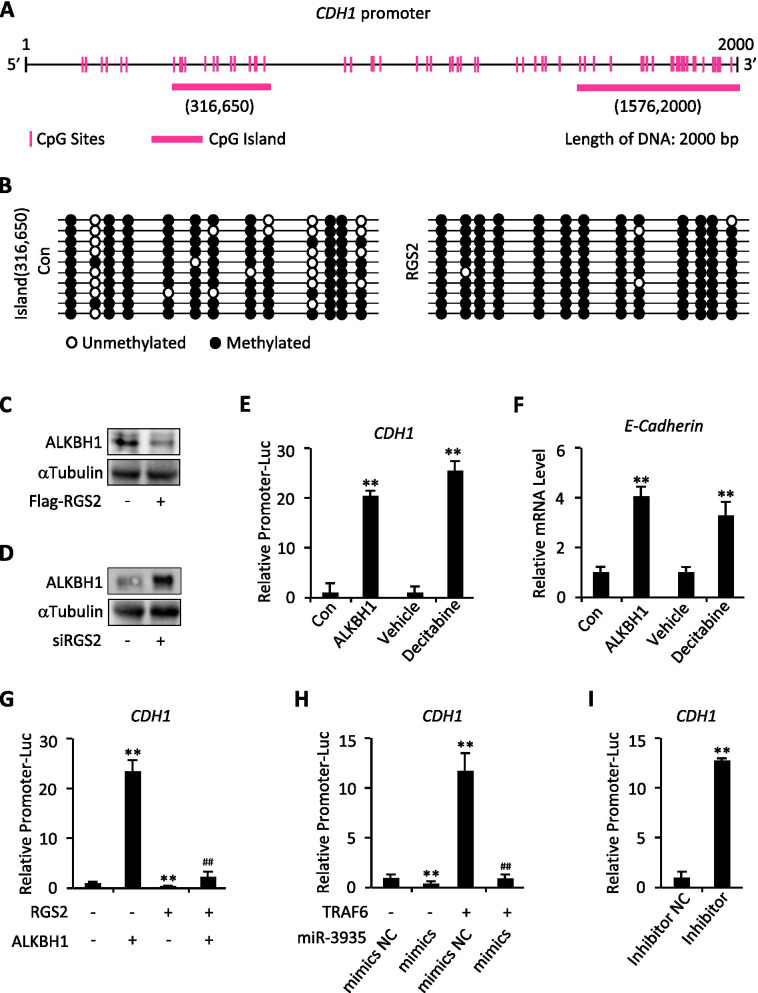
RGS2 regulates *CDH1* promoter methylation. **A** The putative CpG sites within *CDH1* promoter are shown. **B** Methylation status of 11 CpG sites on the promoter region of *CDH1* gene. Methylation analysis was performed in ten clones for each group. Each row of circles represents a single clone, and each circle represents a single CpG site. Open circle represents unmethylated cytosine; filled circle represents methylated cytosine. **C** Western blot analysis for ALKBH1 expression levels in Flag-RGS2-transfected or control vector-transfected (Flag-RGS2, -) HTR-8/SVneo cells. **D** Western blot analysis for ALKBH1 expression levels in RGS2 siRNA-transfected (siRGS2) or control siRNA-transfected (siRGS2, -) HTR-8/SVneo cells. **E** Luciferase assay of HTR-8/SVneo cells co-transfected with firefly luciferase constructs containing the promoter construct of *CDH1* and ALKBH1 or empty vector (Con) or treatment with decitabine for 24 h. **F** qRT-PCR analysis for *E-Cadherin* in HTR-8/SVneo cells transfected with ALKBH1 or treated with decitabine for 24 h. **G** Luciferase assay of HTR-8/SVneo cells co-transfected with firefly luciferase constructs containing the promoter construct of *CDH1* and RGS2 and/or ALKBH1. **H** Luciferase assay of HTR-8/SVneo cells co-transfected with firefly luciferase constructs containing the promoter construct of *CDH1* and miR-3935 mimics and/or TRAF6. **I** Luciferase assay of HTR-8/SVneo cells co-transfected with firefly luciferase constructs containing the promoter construct of *CDH1* and miR-3935 inhibitor or negative control (NC). **p < 0.01 versus Con, vehicle, (RGS2 -, ALKBH1 -), mimics negative control (NC) without TRAF6 or inhibitor negative control (NC); ##*p* < 0.01 versus RGS2 without ALKBH1 or miR-3935 mimics with TRAF6; data represent the mean ± SD of three independent experiments

EMT can be initiated by expression alteration of E-Cadherin [23]. Given that the protein expression of E-Cadherin (encoded by *CDH1* gene) is affected by RGS2 and that the promoter region (~ 2000 bp) of *CDH1* contains two CpG islands with rich CpG sites (Fig. 4D,E and 5A), we supposed that RGS2 might regulate E-cadherin expression by modulation of *CDH1* promoter methylation status. To this end, HTR-8/SVneo cells were transfected with RGS2 or a control empty vector and were cultured for 48 h, followed by examination of the methylation status of the promoter region by means of methylation-specific PCR (MSP) and bisulfate sequencing (BSP). Considering the CpG island that located at (316,650) in *CDH1* gene promoter covered fewer sites (11 CpG sites, Fig. 5A), we thus chose this island for further sequencing. As expected, the CpG island (316,650) in promoter region of *CDH1* was obviously methylated upon RGS2 overexpression, and among the 11 CpG sites tested, particularly the methylation status of site 2 and site 9 within the CpG island were markedly up-regulated, compared with that from control group (Fig. 5B), suggesting the effect of RGS2 on *CDH1* promoter methylation.

It has been reported that the gene expression is associated with the N6-methyladenine DNA modification, and N6-methyladenine levels were dynamically regulated by the DNA demethylase ALKBH1, depletion of which led to transcriptional silencing of genes through decreasing chromatin accessibility [24]. To investigate the potential effect of RGS2 on ALKBH1, we subsequently examined the effect of RGS2 on ALKBH1. Results revealed that overexpression of RGS2 obviously decreased ALKBH1 protein expression, whereas knockdown of RGS2 increased that in HTR-8/SVneo cells (Fig. 5C,D), suggesting the involvement of ALKBH1 in RGS2-mediated *CDH1* promoter methylation. To further confirm ALKBH1 regulation of E-Cadherin expression, we next ectopically expressed ALKBH1 in HTR-8/SVneo cells for further exploration. Overexpression of ALKBH1 not only significantly increased *CDH1*-luciferase activities, but also markedly up-regulated *E-Cadherin* mRNA expression (Fig. 5E,F), which could be additionally verified by decitabine, a DNA methylation inhibitor [25] (Fig. 5E,F). However, overexpression of RGS2 markedly decreased *CDH1*-luciferase activities, which was attenuated by ectopic expression of ALKBH1 (Fig. 5G). Similar to RGS2, overexpression of miR-3935 mimics also dampened *CDH1*-luciferase activities, which could be almost completely restored by TRAF6-overexpression, whereas miR-3935 inhibitor robustly potentiated that (Fig. 5H,I). Thus, RGS2 through ALKBH1 regulates *CDH1* promoter methylation and thereby controls *E-Cadherin* transcription.

### MiR-3935 expression correlates with RGS2 mRNA level in PE

**Fig. 6:**
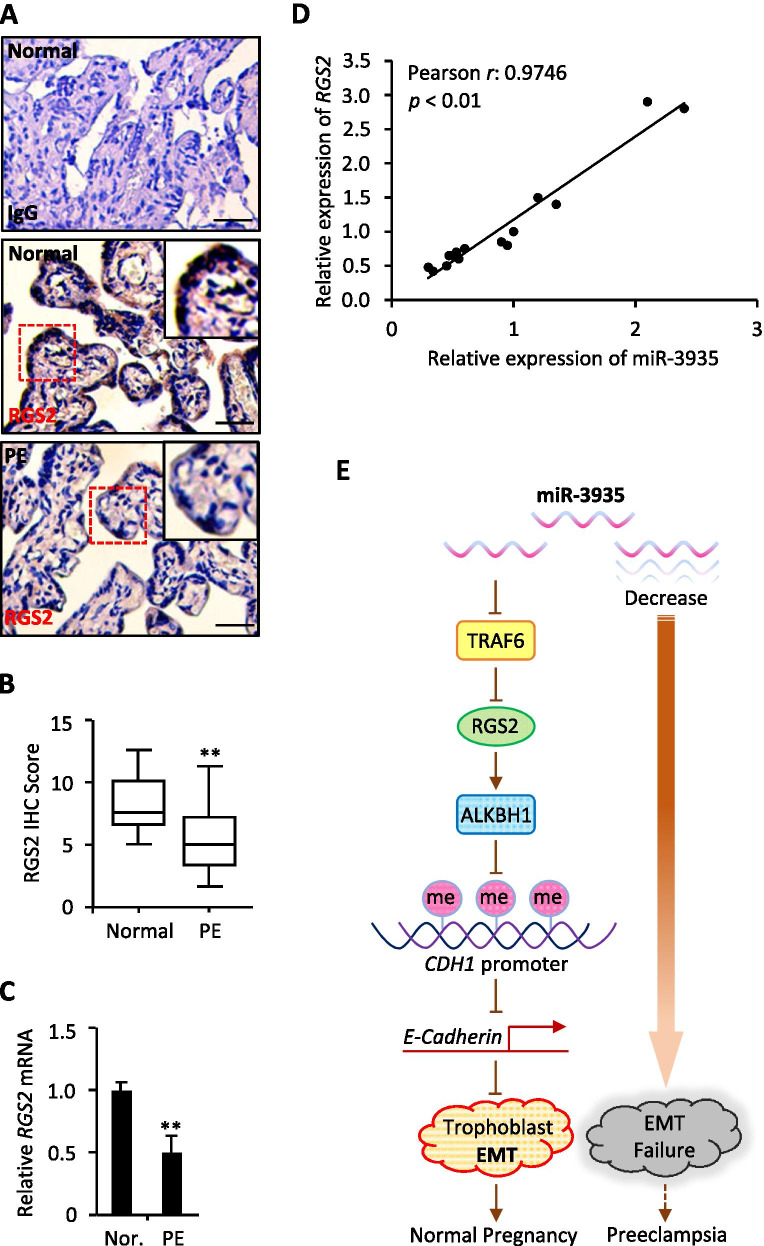
MiR-3935 expression correlates with *RGS2* mRNA level. **A** Immunohistochemistry (IHC) staining for RGS2 by using paraffin-embedded sections of placentas samples from patients with PE and from normal pregnancies. IgG was used as a negative control (up). *N* = 15; bar, 100 μm. **B** IHC score for RGS2 in (**A**). **p < 0.01 versus normal group (Normal). **C** The mRNA expression of *RGS2* was measured using qRT-PCR in placentas samples from 15 patients with PE and 15 women with normal pregnancies. **p < 0.01 versus normal group (Nor.). Data represent the mean ± SD of three independent experiments. **D** The correlation between miR-3935 and *RGS2* in placentas was determined by Pearson’s correlation coefficient (*r*: 0.9746, *p* < 0.01). *N* = 15. **E** A working model for miR-3935/TRAF6/RGS2 signal axis in human trophoblast EMT

To further elucidate the relationship between miR-3935 and RGS2 in PE, we detected the RGS2 expression in 15 paired placentas from patients with PE and women with normal pregnancies. Immunohistochemistry staining showed that levels of RGS2 were lower in PE than that in normal pregnancies (Fig. 6A,B), and qRT-PCR results further revealed that the mRNA expression of RGS2 in PE group was significantly down-regulated when compared with normal group (Fig. 6C). More importantly, the mRNA expression levels of RGS2 in 15 represented normal and PE placentas correlated with the miR-3935 levels (Pearson r: 0.9746, *p* < 0.01, Fig. 6D), which could be also verified in 15 placentas of normal group (Pearson r: 0.902, *p* < 0.01, data not shown) as well as in 15 PE placentas (Pearson r: 0.911, *p* < 0.01, data not shown), respectively. Thus, these data finally indicated that miR-3935 regulates RGS2 expression and their correlation could be determined in both normal and PE clinical samples.

## Discussion

By using biochemical, bioinformatics and clinical approaches, the present study describes, to our knowledge for the first time, the expression and the function of miR-3935 in the development of PE. Our data exhibited that miR-3935 expression was reduced in preeclamptic placentas and peripheral blood specimens. Moreover, we showed that miR-3935 played a key role in trophoblast cells EMT, whose deficiency resulted in PE. At the molecular level, down-regulated expression level of miR-3935 potentiated *TRAF6* transcription, a target of miR-3935, which declined RGS2 expression, leading to the up-regulated ALKBH1 expression and subsequently down-regulated methylation status of *CDH1* gene promoter. As a result, E-Cadherin expression was increased, which thereby suppressed trophoblast cells EMT (Fig. 6E). Our results therefore identified miR-3935 is a novel miRNA that is closely associated with PE development, indicating the critical role of miR-3935 in supporting the physiological function of human placenta, which also provides with the possible mechanisms of miR-3935 regulation in the progression of other human diseases, in addition to PE.

A great deal of research suggests that the expression of miRNAs is deregulated in preeclamptic placentas and the alternation of miRNAs plays an important role in the development and progression of PE [26–28]. In our previous study, we showed that miR-20b was up-regulated in preeclamptic placentas, which inhibited the invasion and migration of trophoblast cells by decreasing MMP-2 expression [16]. Moreover, other miRNAs, such as miR-141 and miR-181a, were also found to be up-regulated in preeclamptic placentas and plasma, indicating their function in PE [29, 30]. In the present study, we demonstrate that miR-3935 is significantly down-regulated in preeclamptic placentas and peripheral blood specimens. Our data thus imply miR-3935 may serve as a serum biomarker for PE, whose abnormal down-regulation may indicate the occurrence of diseases such as PE. Despite the close association between miR-3935 and PE that we found here, it is still uncertain whether down-regulated miR-3935 alone triggers PE, which merits further investigations. Thus, it would be interesting to perform in vivo experiments, such as utilize of miR-3935 knockout transgenic mice (*miR-3935*^*−/−*^) and adenovirus-mediated miR-3935 overexpression in a PE mouse model, to further explore the function of miR-3935 in prevention of PE and the potential therapeutic value of miR-3935 in PE.

Tumor necrosis factor receptor-associated factor 6 (TRAF6), a ubiquitin E3 ligase, participates in the regulation of multiple biological processes, such as exacerbation of pathological cardiac hypertrophy [31], a major predisposing factor for heart failure, arrhythmia and sudden death. In the present study, we provide with evidence that TRAF6 down-regulates RGS2 expression. Regulator of G Protein signaling (RGS) protein family impedes heterotrimeric G protein signaling by regulation of GTPase activity, among which the B/R4 family such as RGS2 is increasingly recognized as a potential contributor to cardiovascular control during pregnancy. A previous report demonstrated that a specific single nucleotide polymorphism (SNP) in the 3’ untranslated region of the *RGS2* gene (rs4606) in mothers is associated with PE [32–34]. Moreover, mutations in *RGS2* gene are associated with human hypertension and reduced RGS2 expression has been demonstrated in hypertensive populations [35–37]. In addition, deletion of *Rgs2* (*Rgs2*^*−/−*^) results in increased resistance and reduced flow in uterine arteries of non-pregnant mice [38]. In our present study, by bioinformatics analysis and luciferase reporter assays, although *RGS2* was not a direct target of miR-3935, a positive correlation was observed in preeclamptic placentas, suggesting down-regulated expression of RGS2 could cause PE, which is consistent with the results from *Katherine* et al. in 2020, showing that RGS2 expression is suppressed in human PE placenta, and that feto-placental disruption of *Rgs2* is sufficient to initiate selected characteristics of PE in pregnant C57BL/6 J mice [39]. However, different from previous study showing *RGS2* transcription is dependent upon HDAC9 (histone deacetylase-9) activity [39], our results revealed that RGS2 mRNA expression is regulated by miR-3935, and inhibition of RGS2 by siRNA could reverse the promoting effect of miR-3935 mimics on trophoblast cells EMT, whereas overexpression of RGS2 could attenuated the negative effects of miR-3935 inhibitors on migration and invasion in trophoblast cells. Thus, our data demonstrates that the RGS2-promoted trophoblastic EMT is at least partly controlled by miR-3935. Despite a previous report showing that RGS2 down-regulation was observed in early prostate cancer (PC) caused by hypoxia and E-cadherin expression was decreased [22], which suggests priming for EMT and is inconsistent with our findings here, high RGS2 levels were correlated to poor patient survival and a positive metastatic status in advanced PC, indicating the different roles of RGS2 between indolent and metastatic forms of cancer and further suggesting that RGS2-related EMT during embryogenesis and carcinogenesis may share different molecular mechanisms.

E-Cadherin expression has been previously reported to be affected by epigenetic alterations such as histone acetylation [40, 41]. Our data of methylation analysis of *CDH1* promoter reveal the presence of rich CpG sites, suggesting that methylation may have a role in shutdown of *CDH1* gene expression. In vertebrates, methylation of cytosine to 5-methylcytosine, which occurs particularly in the context of CpG dinucleotides, establishes a structural substrate for alterations in the structure of chromatin. Cytosine methylation imprints a specific methylation pattern on the DNA sequence and usually serves to properly silence target genes in a tissue-specific manner during embryonic development [42–44]. Different from other studies showing dysregulation of ALKBH1 activity causes many human diseases, including cancer [45], our findings indicate that the precise regulation of ALKBH1 expression is also important and abnormal ALKBH1 expression is associated with deficiency of human trophoblast cells functions such as EMT, which is consistent with a previous report showing *Alkbh1* knockout mice (*Alkbh1*^*−/−*^) resulted in impaired placental trophoblast lineage differentiation [46], suggesting the essential role of ALKBH1 in placentation and embryonic development. Though we show RGS2 regulates ALKBH1 protein expression in the present study, the molecular mechanisms need to be further addressed.

## Conclusions

In the present study, we investigated the roles and underlying mechanisms of miR-3935 in trophoblast EMT by use of the human extra-villous trophoblast cell line HTR-8/SVneo as well as human placental tissues and maternal blood samples. Together our results uncover a hitherto uncharacterized role of miR-3935/TRAF6/RGS2 axis in the EMT function of human trophoblasts, which may pinpoint the molecular pathogenesis of PE and may be a prognostic biomarker and therapeutic target for such obstetrical diseases as PE.

## Data Availability

All data generated or analyzed during this study are included in this published article.

## Abbreviations

BSP: Bisulfate sequencing
DAB: Diaminobenzidine
DAPI: 4',6-Diamidino-2-phenylindole
DMEM: Dulbecco’s modified Eagle’s medium
EMT: Epithelial-mesenchymal transition
EVT: Extra-villous trophoblast
FBS: Fetal bovine serum
GO: Gene ontology
HDAC9: Histone deacetylase-9
HET293T: Human embryonic kidney 293 T
microRNA: MiRNA
MSP: Methylation-specific PCR
NC: Negative control
PE: Preeclampsia
qRT-PCR: Quantitative reverse transcription-PCR
RGS: Regulator of G protein signaling
SNP: Single nucleotide polymorphism
TRAF6: TNF receptor associated factor 6
